# Transcriptomic Analysis of Heat Stress Response in *Brassica rapa* L. ssp. *pekinensis* with Improved Thermotolerance through Exogenous Glycine Betaine

**DOI:** 10.3390/ijms24076429

**Published:** 2023-03-29

**Authors:** Jin Quan, Xinyuan Li, Zewei Li, Meifang Wu, Biao Zhu, Seung-Beom Hong, Jiang Shi, Zhujun Zhu, Liai Xu, Yunxiang Zang

**Affiliations:** 1 Key Laboratory of Quality and Safety Control for Subtropical Fruit and Vegetable, Ministry of Agriculture and Rural Affairs, Collaborative Innovation Center for Efficient and Green Production of Agriculture in Mountainous Areas of Zhejiang Province, College of Horticulture Science, Zhejiang A&F University, Hangzhou 311300, China; 2020601042036@stu.zafu.edu.cn (J.Q.);; 2 Department of Biotechnology, University of Houston Clear Lake, Houston, TX 77058-1098, USA; 3 Institute of Crop Science, Hangzhou Academy of Agricultural Sciences, Hangzhou 310024, China

**Keywords:** glycine betaine, heat stress, *B. rapa*, thermotolerance, protein processing in endoplasmic reticulum, HSP

## Abstract

Chinese cabbage (*Brassica rapa* L. ssp. *pekinensis*) is sensitive to high temperature, which will cause the *B. rapa* to remain in a semi-dormancy state. Foliar spray of GB prior to heat stress was proven to enhance *B. rapa* thermotolerance. In order to understand the molecular mechanisms of GB-primed resistance or adaptation towards heat stress, we investigated the transcriptomes of GB-primed and non-primed heat-sensitive *B. rapa* ‘Beijing No. 3’ variety by RNA-Seq analysis. A total of 582 differentially expressed genes (DEGs) were identified from GB-primed plants exposed to heat stress relative to non-primed plants under heat stress and were assigned to 350 gene ontology (GO) pathways and 69 KEGG (Kyoto Encyclopedia of Genes and Genomes) pathways. The analysis of the KEGG enrichment pathways revealed that the most abundantly up-regulated pathways were protein processing in endoplasmic reticulum (14 genes), followed by plant hormone signal transduction (12 genes), ribosome (8 genes), MAPK signaling pathway (8 genes), homologous recombination (7 genes), nucleotide excision repair metabolism (5 genes), glutathione metabolism (4 genes), and ascorbate and aldarate metabolism (4 genes). The most abundantly down-regulated pathways were plant-pathogen interaction (14 genes), followed by phenylpropanoid biosynthesis (7 genes); arginine and proline metabolism (6 genes); cutin, suberine, and wax biosynthesis (4 genes); and tryptophan metabolism (4 genes). Several calcium sensing/transducing proteins, as well as transcription factors associated with abscisic acid (ABA), salicylic acid (SA), auxin, and cytokinin hormones were either up- or down-regulated in GB-primed *B. rapa* plants under heat stress. In particular, expression of the genes for antioxidant defense, heat shock response, and DNA damage repair systems were highly increased by GB priming. On the other hand, many of the genes involved in the calcium sensors and cell surface receptors involved in plant innate immunity and the biosynthesis of secondary metabolites were down-regulated in the absence of pathogen elicitors in GB-primed *B. rapa* seedlings. Overall GB priming activated ABA and SA signaling pathways but deactivated auxin and cytokinin signaling pathways while suppressing the innate immunity in *B. rapa* seedlings exposed to heat stress. The present study provides a preliminary understanding of the thermotolerance mechanisms in GB-primed plants and is of great importance in developing thermotolerant *B. rapa* cultivars by using the identified DEGs through genetic modification.

## 1. Introduction

Heat stress is one of the most common risks that threatens plant growth and development in the current global warming environment [1]. It generally causes plant metabolic imbalance, cytoskeleton dismantling, and oxidative damage, which can lead to plant wilting and death [1,2,3]. The main effects of heat stress are denaturation of heat-labile proteins and accumulation of harmful reactive oxygen species (ROS) in plant cells [4,5]. To protect from such heat-induced damages, plants have evolved a repertoire of heat shock proteins (HSPs). Heat shock factors (HSFs) play a key role in coping with heat stress through the regulated expression of HSPs and other stress-associated proteins that mitigate high temperature effects [6]. HSP70 is considered to be the most abundant protein produced in response to heat stress [7,8]. It acts as a molecular chaperone that protects cellular proteins from irreversible denaturation and stabilizes the cell membrane and cytoskeleton [9].

The increase in flavonoid substances derived from phenylpropanoid metabolic pathway in some adult plant species was shown to enhance their ability to resist biotic and abiotic stresses [10,11,12,13]. Due to the antioxidant properties of polyphenols [14], accumulation of various phenolic compounds potentially contributes to scavenging excessive ROS generated from external stresses [15]. Heat stress also increased the accumulation of phenolics in tomato [16,17] and expression of the genes involved in oxylipin and proline biosynthesis in creeping bentgrass [18]. As a result, phenolic accumulation is usually a dependable feature exhibited by plants under stress.

Glycine betaine (GB) is a quaternary ammonium compound that plays a protective role in plant response to abiotic stresses [19]. As a zwitterionic osmolyte, this low molecular weight compound can interact with both hydrophilic and hydrophobic domains of protein complexes and membranes. This helps to maintain the structural and functional integrity of those macromolecules [20,21,22]. In addition, exogenous GB attenuated oxidative stress by increasing higher levels of both enzymatic and non-enzymatic antioxidants in plants [23,24]. It is now widely regarded as a plant biostimulant [25,26] and is commonly found in haloarchaea, bacteria, marine invertebrates, and animals. However, only a few crop plants synthesize and accumulate GB [20,24]. While some higher plants significantly accumulate GB in response to environmental stresses, its accumulation can also be cultivar or genotype-specific [27]. GB is synthesized from choline via two consecutive oxidation reactions in chloroplast stroma: choline monooxygenase (CMO) catalyzes oxidation of choline to betaine aldehyde (BA), which is then oxidized to yield GB by BA dehydrogenase (BADH) [28,29]. The GB can be translocated throughout the plant via the phloem [30]. Thus, increased levels of GB by enhanced activities of both CMO and BADH can reduce excitation pressure and redox imbalance resulting from excessive photosynthetic electrons produced by stresses [17,27]. Exogenous application of abscisic acid (ABA), which plays a key role in stress tolerance, substantially increased the BADH mRNA levels in barley and sorghum plants able to synthesize GB [31,32]. GB works as a plant defense-priming molecule capable of inducing tolerance to a wide range of biotic and abiotic stresses by conferring the osmoprotection and thermodynamic stability of various macromolecules, as well as by reversing the misfolded and/or aggregated proteins to restore their functional activities [24,33,34]. GB can also enhance photosynthesis by protecting the ribulose-1,5-bisphosphate carboxylase/oxygenase (Rubisco) and photosystem II during heat stress [35].

*B. rapa*, an economically important vegetable crop originating in China, is widely cultivated in many other countries [36]. Like *Arabidopsis*, *B. rapa* is not able to synthesize GB [20,37,38,39]. As a cool-weather crop, *B. rapa* is susceptible to heat, especially in early August in the high temperature (over 30 °C) of the Yangtze River area in China [40]. Previously we reported that GB priming substantially enhanced the thermotolerance of *B. rapa* seedlings by promoting photosynthesis performance, osmoprotection, and antioxidant enzyme activity [41]. To further gain an insight into the underlying molecular mechanisms of GB-primed thermotolerance, we investigated the genome-wide gene expression profiles of *B. rapa* seedlings treated with GB before heat stress.

## 2. Results

### 2.1. Effects of GB on RWC in B. rapa under Heat Stress

**Figure 1:**
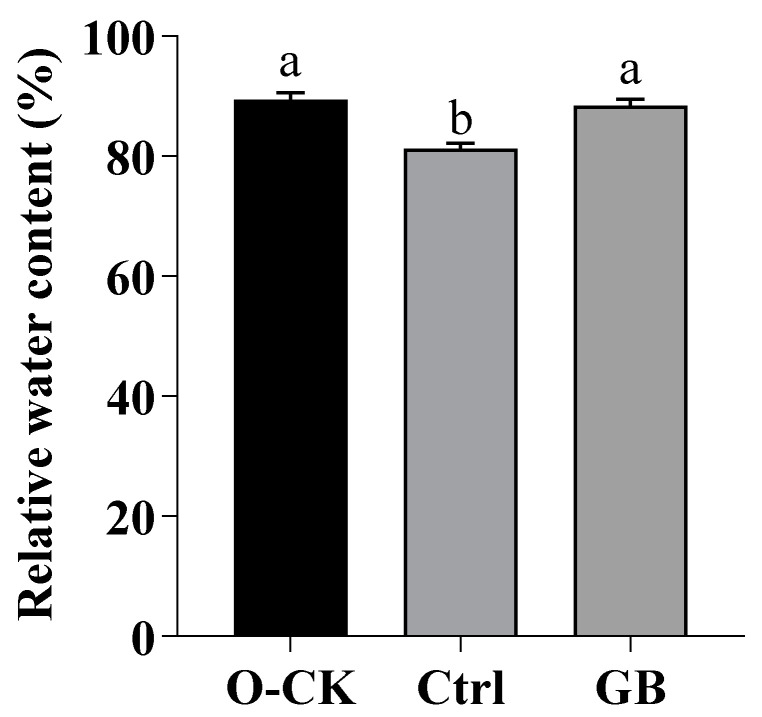
Effects of GB on relative water content (RWC) in *B. rapa* under heat stress. Data are shown as mean ± SD. Different lowercase letters indicate significant difference at *p* < 0.05. O-CK, optimum conditions, foliar sprayed with water; Ctrl, foliar sprayed with water before exposure to heat stress; GB, foliar sprayed with GB before exposure to heat stress.

High temperature generally increases water loss and may thus cause internal water deficit. Heat tolerance may depend on plant water status. RWC, which reflects the balance between water supply to the leaf tissue and transpiration rate, has previously been reported as a good indicator for dehydration stress [42,43]. Upon GB application, RWC was significantly increased by 8.84%, as compared to the heat-stressed control, but no significant difference in RWC was noted between GB treatment and optimal conditions control (Figure 1). This result implicated that GB enhanced the capacity of osmotic adjustment even under heat stress.

### 2.2. Effects of GB on O_2_^·−^in B. rapa under Heat Stress

**Figure 2:**
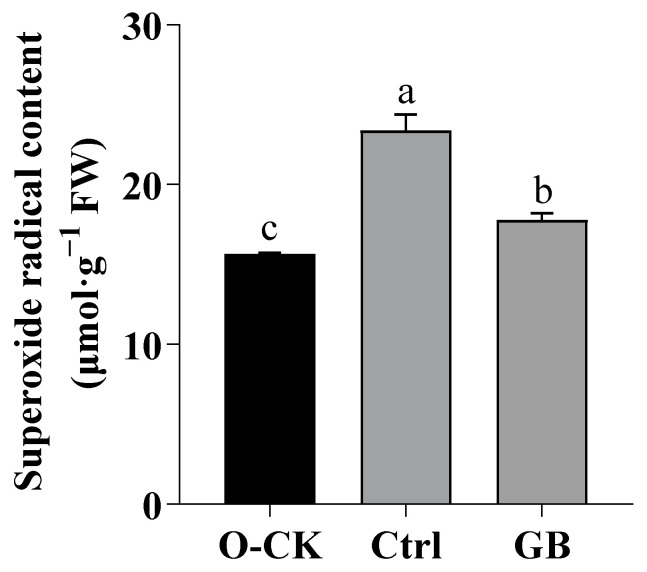
Effects of GB on O_2_^·−^ in *B. rapa* under heat stress. Data are shown as mean ± SD. Different lowercase letters indicate significant difference at *p* < 0.05. O-CK, optimum conditions, foliar sprayed with water; Ctrl, foliar sprayed with water before exposure to heat stress; GB, foliar sprayed with GB before exposure to heat stress.

When the plants suffer from heat stress, the balance of reactive oxygen radicals is disrupted as a result of excessive ROS accumulation. Activation of the ROS scavenging system helps to reduce the oxidative damage [44,45]. Under heat stress, O_2_^·−^ increased by 49.44% compared with *B. rapa* of optimum conditions (Figure 2). In GB-primed plants exposed to heat stress, O_2_^·−^ significantly decreased by 31.84% relative to heat stress control. Thus, GB treatment activated the plant ROS scavenging system.

### 2.3. Effects of GB on ATP Content in B. rapa under Heat Stress

**Figure 3:**
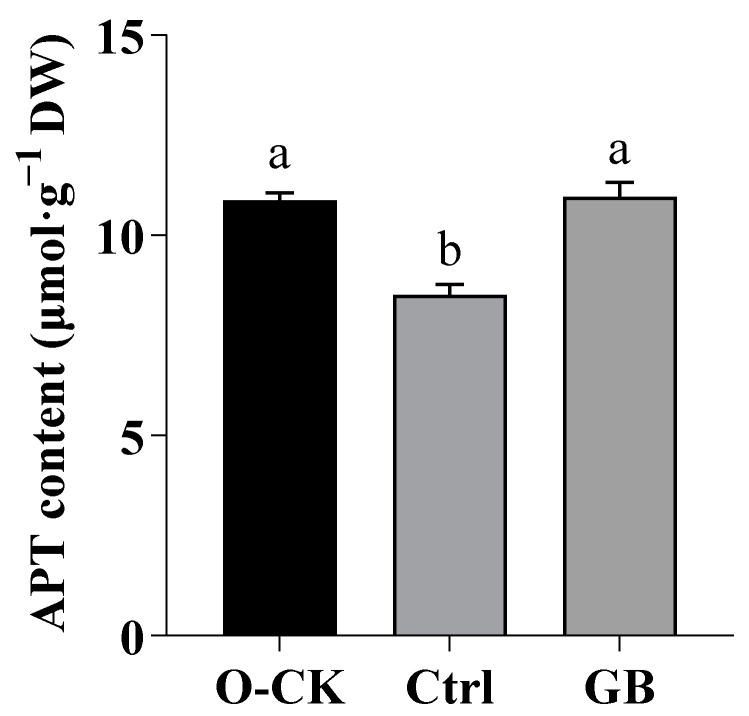
Effects of GB on ATP content in *B. rapa* under heat stress. Data are shown as mean ± SD. Different lowercase letters indicate significant difference at *p* < 0.05. O-CK, optimum conditions, foliar sprayed with water; Ctrl, foliar sprayed with water before exposure to heat stress; GB, foliar sprayed with GB before exposure to heat stress.

ATP is a suitable biomarker to address cellular stress response because additional energy costs are incurred by the mechanisms imparting thermotolerance [46]. As shown in Figure 3, the ATP content of *B. rapa* decreased under heat stress by 27.78%, compared with optimum conditions. However, GB priming remarkably improved ATP by 28.67%, compared to the heat-stressed control. There was no significant difference between optimum conditions and exogenous GB treatment under heat stress. This suggested that exogenous GB enhanced photosynthesis and cellular respiration to a level being comparable to optimum conditions.

### 2.4. Transcriptomic Profiling of B. rapa in Response to Heat Stress

In order to reveal the molecular responses of GB-primed thermotolerance, six cDNA libraries were constructed from *B. rapa* seedling leaves pretreated with or without GB under heat stress and sequenced. A total of 52.24 Gb clean data were obtained, and the amount of clean data of each library reached 6.24 Gb, with a Q30 base percentage greater than 91.42%. The sequence alignment efficiency of clean reads of each library against the designated reference genome ranged from 81.65% to 86.88% (Table S1). A total of 351,258,726 Mb reads were obtained from six samples, with an average of 58,543,121 Mb reads in each sample (Table S2). Approximately 82.91% of Ctrl and 80.88% of GB reads were aligned uniquely with the reference genome.

**Figure 4:**
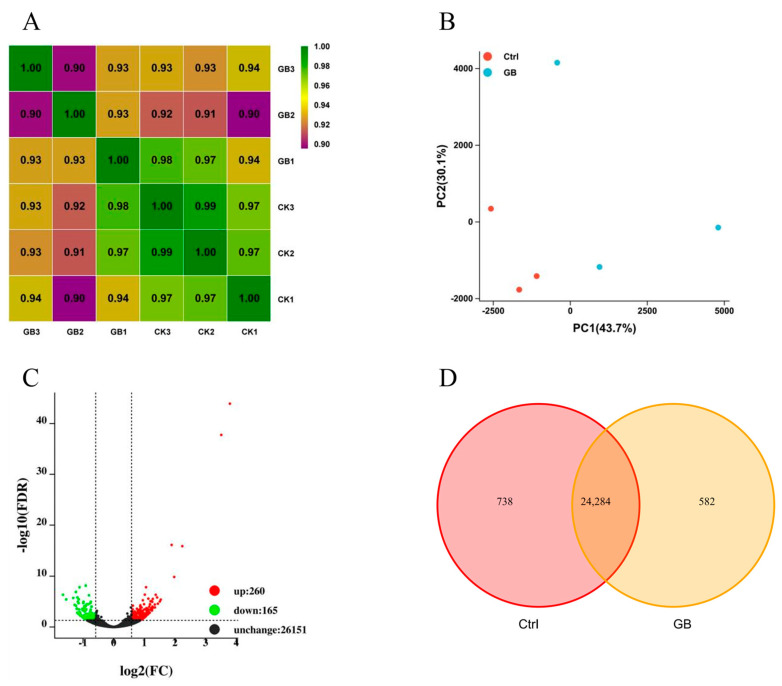
RNA-Seq data analyses. (**A**) Heatmap of correlations between samples in different treatments showing three GB and three control (CK) groups. Each value in the heatmap is a Pearson’s correlation coefficient. (**B**) PCA analysis plot displaying all 6 samples along the axes of PC1 and PC2, which describe 43.7% and 30.1% variability, respectively, within the expression dataset. (**C**) Volcano plots of the overall DEGs distribution. Threshold was set as an adjusted *p*-value (Padj) < 0.05 in GB relative to Ctrl. X-axis depicts fold change (FC) in gene expression where the smaller the value, the higher the expression relative to Ctrl. Y-axis depicts false statistical significance of false discovery rate (FDR), where the bigger the value, the smaller the ratio of the number of false positives to the number of total positives. Significantly up-regulated and down-regulated genes are indicated in red and green, respectively. Genes that were not differently expressed are depicted in black. (**D**) Venn diagram of expressed genes in GB and control under heat stress.

The heat map proved good repeatability (intragroup variability) and reproducibility (intergroup variability) in the datasets (Figure 4A). Principal component analysis (PCA) showed that the replicate data in the Ctrl and GB treatments were obviously separated in the PC2 dimension (Figure 4B). Differentially expressed genes (DEGs) were determined by pairwise comparison of the expression data. As compared with Ctrl, exogenous GB significantly up-regulated 260 DEGs and down-regulated 165 DEGs (Figure 4C).

The Venn diagram shows that there was a total of 25,022 and 24,866 genes in Ctrl and GB, respectively (Figure 4D). Among the identified genes, 738 and 582 DEGs were specifically expressed in Ctrl and GB, respectively.

### 2.5. GO and KEGG Pathway Analysis of DEGs

**Figure 5:**
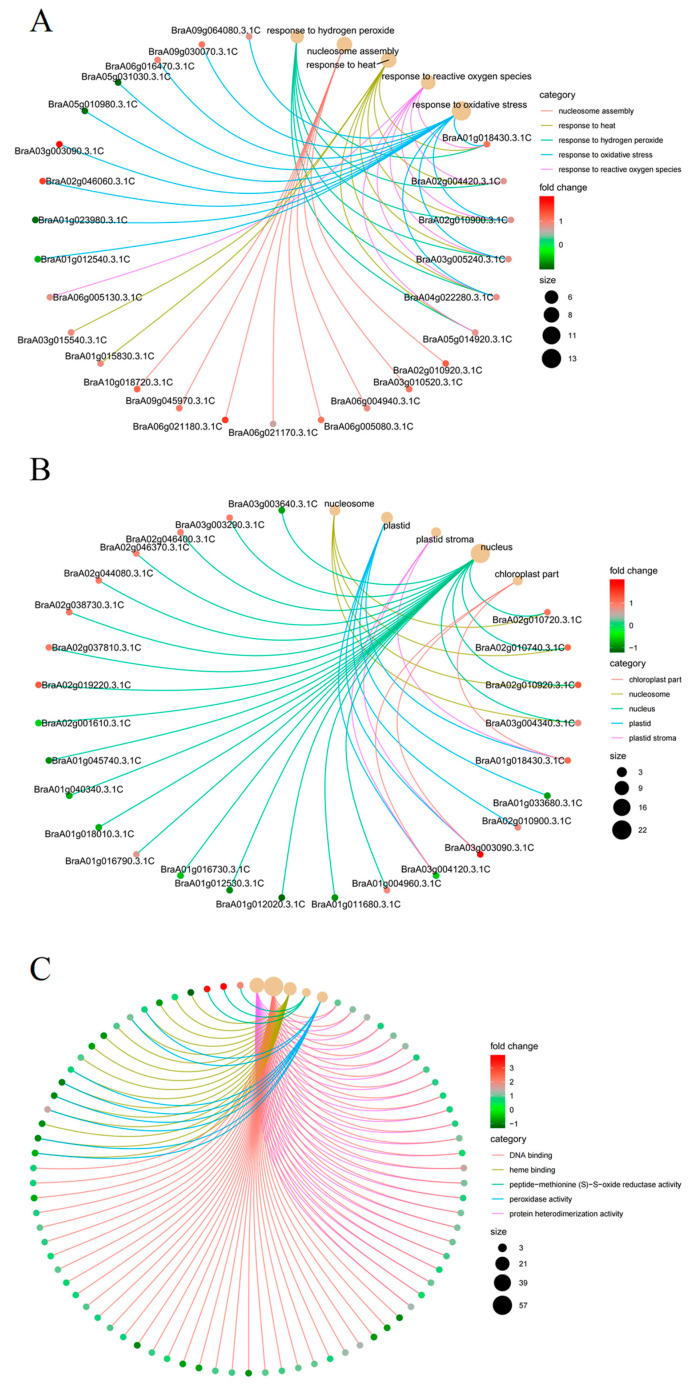
GO enrichment analysis. (**A**) BP, biology process; (**B**) CC, cell component; (**C**) MF, molecular function. The size and color of the point indicates the number of differentially expressed genes in the pathway and the fold change as the threshold for significantly differential expression, respectively.

To identify the function of the sets of genes impacted, we conducted GO enrichment analysis of DEGs in GB relative to Ctrl (Figure 5, Table S3). In total, 350 DEGs were successfully classified into the biological process (BP), cellular component (CC), and molecular function (MF). The highly enriched BP terms were response to heat (GO:0009408), response to high light intensity (GO:0009644), response to hydrogen peroxide (GO:0042542), response to reactive oxygen species (GO:0000302), and response to oxidative stress (GO:0006979) (Figure 5A). The significantly enriched CC terms were nucleus (GO:0005634), nucleosome (GO:0000786), plastid (GO:0009536), plastid stroma (GO:0009532), and chloroplast part (GO:0044434) (Figure 5B). Within the MF terms, DNA binding (GO:0003677), protein heterodimerization activity (GO:0046982), peptide-methionine (S)-S-oxide reductase activity (GO:0008113), protein self-association (GO:0043621), and unfolded protein binding (GO:0051082) were highly enriched (Figure 5C). The results indicated that GB coordinately regulated various biological pathways to trigger plant adaptation to heat stress.

**Figure 6:**
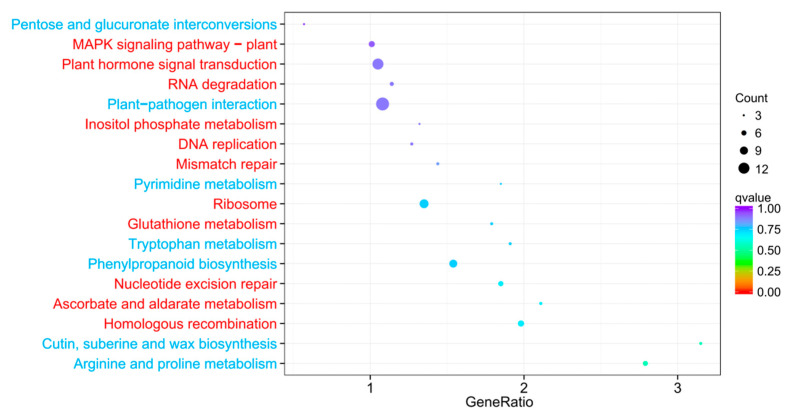
KEGG pathway enrichment analysis. The vertical axis represents the pathway name, and the horizontal axis represents the gene number ratio of significant DEGs to the total genes in a given KEGG pathway. The size of the dots indicated by “Count” represents the number of DEGs in the pathway, and the color of the dots corresponds to the different q-value ranges. The figure shows the top 18 pathways with q-values. Red and blue letters indicate more than 50% up-regulated and down-regulated DEGs out of a total number of DEGs, respectively.

The KEGG pathway enrichment analysis was conducted to systematically analyze the metabolic pathways of DEGs. There were 82 KEGG pathways enriched in GB relative to Ctrl (Table S4). Based on the values of corrected -log10 (Padj) and GeneRatio, 30 of the most enriched KEGG pathways were selected, in which 16 pathways contained relatively more up-regulated genes, and 12 pathways comprised more down-regulated genes (Figure 6, Table S4). The most abundantly up-regulated pathway was protein processing in endoplasmic reticulum (14 genes) in which 12 genes were up-regulated and 2 genes were down-regulated by GB priming. This was followed by plant hormone signal transduction (12 genes), ribosome (10 genes), MAPK signaling pathway (8 genes), homologous recombination (7 genes), nucleotide excision repair metabolism (6 genes), RNA degradation (5 genes), DNA replication (4 genes), mismatch repair (4 genes), glutathione metabolism (4 genes), ascorbate and aldarate metabolism (4 genes), inositol phosphate metabolism (3 genes), and pyrimidine metabolism (3 genes). The most abundantly down-regulated pathway was plant-pathogen interaction (14 genes) in which 8 genes were down-regulated and 6 genes were up-regulated by GB priming. This was followed by phenylpropanoid biosynthesis (9 genes); arginine and proline metabolism (6 genes); cutin, suberine, and wax biosynthesis (4 genes); tryptophan metabolism (4 genes); and pentose and glucoronate interconversion (3 genes), (Table S4).

### 2.6. Validation of DEGs in Response to GB Treatment Using qRT-PCR

**Figure 7:**
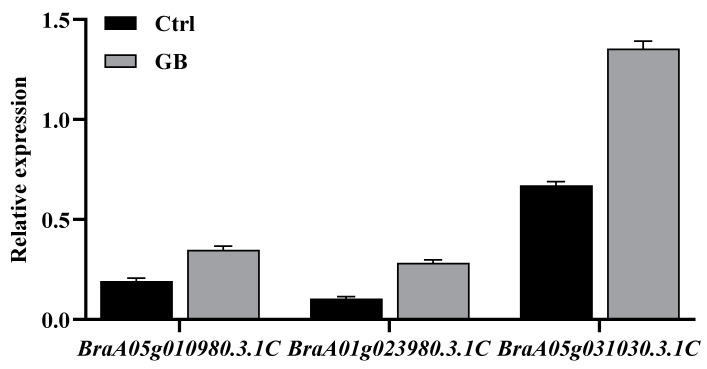
Gene expression pattern verification by qRT-PCR. A. Three genes for HSP20: *BraA03g005240.3.1C*, 17.6 kDa class II heat shock protein; *BraA01g018430.3.1C*, heat shock protein 21 (chloroplastic); *BraA01g015830.3.1C*, 23.6 kDa heat shock protein (mitochondrial). B. Three genes for antioxidant enzymes: *BraA05g010980.3.1C*, peroxidase 19; *BraA01g023980.3.1C*, peroxidase 34; *BraA05g031030.3.1C*, peroxidase 29 C. Three genes of other pathway genes: *BraA08g025430.3.1*, glucose-6-phosphate 1-dehydrogenase 3 (chloroplastic); *BraA03g050640.3.1C*, 5’-adenylylsulfate reductase 3 (chloroplastic); *BraA04g029450.3.1C*, abscisic acid receptor PYL6.

In order to validate our transcriptome data, qRT-PCR was performed on 9 DEGs in the KEGG pathways (Figure 7). HSPs constitute the first line of protection for cells exposed to a variety of stressful conditions by reversing unfolding or inhibiting denaturation and aggregation of cellular proteins as molecular chaperones. We found 6 genes related to HSP from the RNA-seq dataset. There were genes for 3 heat shock proteins and 3 antioxidant enzymes, which were *BraA03g005240.3.1C*, *BraA01g018430.3.1C*, *BraA01g015830.3.1C*, *BraA05g010980.3.1C*, *BraA01g023980.3.1C*, and *BraA05g031030.3.1C*. Three genes (*BraA08g025430.3.1C*, *BraA03g050640.3.1C,* and *BraA04g029450.3.1C*) appeared many times in carbon metabolism, pentose phosphate pathway, glutathione metabolism, sulfur metabolism, MAPK signaling pathway-plant, and plant hormone signal transduction pathway. The results of qRT-PCR showed a similar expression profile to those from the RNA-Seq data (Table S5). These results independently confirmed that high coverage of transcriptome data is reliable.

## 3. Discussion

### 3.1. GB Priming Activated the Genes for Ascorbate-Glutathione Cycle and Deactivated the Genes for Proline Biosynthesis in B. rapa Seedlings under Heat Stress

High temperatures evoke an explosive generation of ROS, causing severe damages to a range of cellular components. Hence, plant adaptation to heat stress would be dependent on simultaneous multiple responses associated with changes in physiological, biochemical, and molecular processes as a result of well-coordinated interplays of many different multigenic protective pathways and complex regulatory networks. In our previous study, we showed that GB, when exogenously applied, enhanced the thermotolerance of Chinese cabbage through the higher levels of photosynthesis, osmoprotection, and antioxidant enzyme activities [41]. This finding is further corroborated by the present study showing that GB pre-treatment increased the contents of RWC and ATP along with the decreased level of superoxide. To attenuate oxidative damage primarily caused by high temperatures, cells must produce higher levels of antioxidant molecules. Our KEGG enrichment results showed that GB priming positively influenced the genes involved in the metabolism of “glutathione”, “ascorbate and aldarate”, and “inositol phosphate” (Table S4). APX1 genes (*BraA06g005130.3.1C*; *BraA09g064080.3.1C*) encoding L-ascorbate peroxidase, which is a H_2_O_2_-scavenging enzyme associated with ascorbate-glutathione cycle, were up-regulated. This redox cycle is known to exist in at least four different subcellular locations, including the cytosol, chloroplast, mitochondria, and peroxisome [47]. In addition, IOX genes (*BraA07g000970.3.1C*; *BraA09g012490.3.1C*) encoding an inositol oxygenase were up-regulated. *IOX* is the first enzyme in the biosynthesis pathway from inositol to ascorbate, which contributes to abiotic stress tolerance by scavenging reactive oxygen species (ROS) [48]. Up-regulation of *P3-G6PDH* (*BraA08g025430.3.1C*) encoding chloroplastic glucose-6-phosphate 1-dehydrogenase was notable because P3-G6PDH catalytic reaction provides NADPH that can play a vital role in protecting cells from oxidative stress because redox reactions involving NADPH produce compounds that prevent ROS [49]. *GGCT* gene (*BraA09g005730.3.1C*) encoding γ-glutamylcyclotransferase that degrades glutathione to its component amino acids L-glutamate, L-cysteine, and L-glycine [50] was up-regulated. This would be needed to sustain the higher activity level of chloroplastic *G6PDH* because it is inactivated by glutathione [51]. In vitro studies have demonstrated that GB, by itself, does not have antioxidative activity [52]. Thus, increased antioxidant activity triggered by GB appears to be ascribed to the coordinated and concerted action of *APX1*, *IOX*, *P3-G6PDH*, and *GGCT* that would facilitate the efficient counteraction of the negative effects of ROS in the acquisition of thermotolerance.

The KEGG enrichment results linked with “arginine and proline metabolism” showed two up-regulated DEGs and two down-regulated DEGs with regard to proline homeostasis. Two up-regulated genes (*BraA02g040640.3.1C*; *BraA06g037190.3.1C*) encode a proline dehydrogenase 1 (PRODH1) that degrades proline. *PRODH1* gene was strongly induced by proline and was assumed to play a role in oxidizing excess proline and transferring electrons to the respiratory chain [53]. Among the down-regulated genes were *P5CSA* (*BraA04g028960.3.1C*; *BraA05g006220.3.1C*), which encodes a bifunctional delta-1-pyrroline-5-carboxylate synthase A that catalyzes the first two steps in proline biosynthesis from glutamate in plants [54]. Thus, up-regulated *PRODH1* and down-regulated *P5CSA* would exert a synergistic effect on removing excessive proline in plants, despite its role as an effective osmo-protective agent and hydroxyl radical scavenger [52]. This result apparently contradicts with the previous finding that heat stress triggered the enhanced expression of the *P5CSA* gene in creeping bentgrass [18]. The discrepancy could be ascribed to the difference in stage of growth and development of plants subjected to heat stress (young seedling versus adult), and/or heat stress conditions (45 °C for 5 days versus 35/30 °C for 21 days). Perhaps cells in the presence of GB and other compatible osmolytes would need to avoid the negative physiological consequences of diverting the precursor to proline away from primary metabolism [55]. Taken together, plant cells appear to possess several different osmoregulation and ROS scavenging systems whose expression can be flexibly changed depending on the stage of growth and development as well as duration and severity of stress conditions.

### 3.2. GB Priming Activated DNA Damage Repair and Heat Shock Response Systems in B. rapa Seedlings under Heat Stress

Sessile plants are constantly exposed to environmental stresses that cause a rapid accumulation of unbalanced levels of ROS able to alter normal cell metabolism. In order to ensure cell integrity and function, cells must adapt their physiological metabolism, which enables cells to protect essential intracellular constituents from external stresses. Above all, activation of signaling pathways devoted to DNA damage repair is a prerequisite for plant survival and productivity. In particular, the capacity of cells to promptly sense and repair DNA damage is essential for preserving cell integrity because heat stress not only inhibits DNA repair systems, but also directly induces single- and double-stranded DNA breaks [56]. According to the literatures, changing temperatures are assumed to be sensed in at least four different ways depending on the degree of temperatures: (i) calcium influx channels [57], (ii) histone H2A.Z-containing nucleosome [58], (iii) regulated intramembrane proteolysis in the endoplasmic reticulum [59,60], and (iv) misfolded protein accumulation in the cytosol [61]. Our KEGG analysis showed that DEGs involved in “DNA replication,” “metabolism of purine and pyrimidine,” and all major types of DNA damage repair including “homologous recombination,” “nucleotide excision,” “base excision,” and “mismatch repair” were up-regulated upon GB treatment. Notable DEGs were *RPA* genes (*Bra03g003290.3.1C*; *Bra09g006930.3.1.C*) encoding replication protein A 70 kDa DNA-binding subunit B and D, *PCNA* gene (*BraA09g064370.3.1C*) encoding proliferating cell nuclear antigen, and *RFC* gene (*BraA09g013330.3.1C)* encoding replication factor C (Table S4). *RPA* plays essential roles in almost all DNA metabolic pathways, including DNA replication, transcription, recombination, cell-cycle checkpoints, and all major types of DNA damage repair, including base excision, nucleotide excision, mismatch, and double-strand break repair [62]. PCNA is a processivity factor for DNA polymerase δ and ɛ, important for DNA replication, DNA repair, and cell cycle regulation [63,64,65]. RFC is a DNA polymerase accessory protein whose function is to load PCNA onto primed DNA templates [66]. Accordingly, elevated expression of the intricate, well-coordinated DNA repair system comprising *RPA*, *PCNA*, and *RFC* would contribute to maintaining genomic integrity during replication in the stress conditions.

The KEGG enrichment results showed that all 10 DEGs linked with “ribosome” and all 14 DEGs linked with “protein processing in endoplasmic reticulum” were up-regulated (Table S4). This implicates the active synthesis of polypeptides by ribosome and quality control process in endoplasmic reticulum where nascent polypeptides are subjected to protein folding and posttranslational modifications for functional proteins [67]. The former encoded 40S and 60S ribosomal protein subunits. The latter comprised ATP-independent class I and II small heat shock proteins (eight sHSPs) and cytosolic, chloroplastic, and mitochondrial ATP-dependent HSPs (HSP70-5 and HSP90-2). Under stress challenges, sHSPs are assumed to seize misfolded proteins to avoid their aggregation and then transfer them to ATP-dependent HSP70s for renaturation [68]. They are all known to be important for basal and acquired thermotolerance [69]. HSPs are critical effectors of the adaptive response to a variety of stresses because HSPs are associated with the proteins involved in the DNA repair signaling pathway [70]. Hence, as the first line of protection, increased expression of HSPs/sHSPs combined with the osmoprotection and ROS scavenging ability of GB would facilitate plant cells to adapt to high temperature or other abiotic stresses.

### 3.3. GB Priming Co-Regulated ABA and Cytokinin-Mediated Signaling Crosstalk Networks to Balance Growth and Stress Responses in B. rapa Seedlings under Heat Stress

A proper plant defense response under stress conditions requires whole plant adaptation and sustained growth. This necessitates co-regulation of the interconnected hormone signaling crosstalk networks at multiple levels to fine-tune stress responses during plant growth and development through both antagonistic and synergistic interaction [71]. According to the KEGG functional enrichment results, GB positively affected “plant hormone signal transduction” and the “MAPK signaling pathway.” The relevant DEGs involved in these pathways were *PLY4 (BraA03g020020.3.1C)*, *PLY6 (BraA04g029450.3.1C)*, *PP2Cs (BraA01g016730.3.1C*; *BraA07g038060.3.1C)*, *ABI5 (BraA05g009100.3.1C)*, *GH3 (BraA09g010480.3.1C)*, *TIR1 (BraA07g026070.3.1C)*, *CAT3 (BraA06g016470.3.1C)*, *NPR2 (BraA01g016790.3.1C)*, *bHLH90 (BraA06g007510.3.1C)*, and *HHO6 (BraA05g020120.3.1C)* (Table S4). *PP2Cs* encode type-2C protein phosphatases, which are known to be key negative regulators of ABA signaling through interaction with the ABA receptor and play an important role in response to various stresses [72]. *PLY4* and *6* genes encode a component of the ABA receptor that inhibits *PP2Cs* [73,74]. Notably, *PLY4* and *6* genes were up-regulated while *PP2Cs* were down-regulated. The coordinated expression control of *PLYs* and *PP2Cs* by GB priming, leading to a synergistic positive action of ABA signaling and response pathway, would trigger ABA-mediated plant adaptation to heat stress because ABA has been found to be an important plant hormone involved in the regulation of plant development and stress responses [74]. *ABI5 (ABA INSENSITIVE 5*), encoding a transcription factor that prevents seed germination and post-germinative seedling growth under unfavorable stress conditions [75], was down-regulated, and thus down-regulation of *ABI5* gene would promote seedling growth under heat stress. This is consistent with the previous findings that exogenous GB enhanced thermotolerance during seed germination and growth of barley and tomato seedlings under heat stress [76,77]. ABI5 was shown to play a crucial role in regulating processes essential for plant adaptation to stress at different developmental stages [75]. GB priming up-regulated *CAT3* gene for catalase that decomposes H_2_O_2_ to water; transcription of *CAT3* was found to be mainly enhanced by ABA and oxidative treatments [78]. Given the function of hydrogen peroxide as a key redox signaling molecule regulating gene expression and hypersensitive cell death [79,80], it would be important to sustain hydrogen peroxide homeostasis for plant survival under heat stress.

*CKX3* gene (*BraA02g012830.3.1C*) linked with “zeatin biosynthesis”, which encodes cytokinin dehydrogenase 3 catalyzing degradation of cytokinin [81], was up-regulated, suggesting its potential role in heat stress conditions and regulatory mechanisms of cytokinin homeostasis. It has been known that cytokinin and ABA have antagonistic functions in diverse physiological processes, including stress tolerance [82]. Cytokinin was shown to facilitate degradation of the ABA signaling component transcription factor *ABI5* [83]. In our study, the *ABI5* gene was down-regulated. Furthermore, transgenic plants with inactivated components of cytokinin signaling pathways or reductions in pools of active cytokinin exhibited increased tolerance to high temperatures [84]. As a result, up-regulation of *CKX3* and down-regulation of *ABI5* would have a synergistic positive effect on the growth of seedlings withstanding heat stress. In plants, UDP-glycosyltransferases (UGTs) glycosylate various phytohormones and other metabolites in response to a variety of biotic and abiotic stresses [85]. For instance, constitutive ectopic expression of *Arabidopsis* glycosyltransferase UGT85A5 enhanced salt stress tolerance in transgenic tobacco [86]. In our KEGG pathways analysis, UDP-glycosyltransferases encoded by *UGT85A2 (BraA09g041870.3.1C)* linked with “zeatin biosynthesis” and *UGT87A2 (BraA03g016060.3.1C)* linked with “tryptophan metabolism” were up-regulated and down-regulated, respectively. *UGT85A2* was shown to be expressed mainly in the areas of active cell division in *Arabidopsis* [87]. The reduced expression of *B. rapa UGT87A2* contrasts with the *Arabidopsis UGT87A2*, which was induced by abiotic stresses and ABA [88]. This discrepancy is likely due to the difference in growth stage of the plants under stress conditions because the expression of *UGT87A2* is developmentally regulated [89]. Taken together, gene expression patterns suggested that GB can modify the production of the endogenous hormones involved in stress responses through the regulated expression of the genes encoding hormone modification or degradation enzymes in an age-dependent manner. 

### 3.4. GB Priming Down-Regulated the Genes for the Biosynthesis of Secondary Metabolites in B. rapa Seedlings under Heat Stress

The majority of DEGs in the KEGG categories of “phenylpropanoid biosynthesis”; “cutin, suberine, and wax biosynthesis”; “tryptophan metabolism”; “arginine and proline metabolism”; and “pentose and glucuronate interconversions” in the KEGG enrichment results were down-regulated upon GB pre-treatment. Most of those down-regulated genes encode the proteins involved in the biosynthesis of secondary metabolites: indole glucosinolate O-methyltransferase 4 (*BraA01g006170.3.1C*) catalyzing methylation of indole glucosinolate compounds [90], class III peroxidases (POX19, 29, 34, 42) catalyzing reduction of peroxides [91], cinnamoyl-CoA reductase 1 (*BraA09g063390.3.1C*) catalyzing the first step of lignin biosynthesis pathway [92], BAHD acyltransferase BIA1-like (*BraA06g025750.3.1C*) catalyzing inactivation of brassinosteroid (BR) hormones [93], At3g29680-like BAHD acyltransferase (*BraA08g022960.3.1C*) encoding anthocyanin acyltransferase that increases pigment stability [94], very-long-chain aldehyde decarbonylase (*BraA03g012310.3.1C*) involved in cuticle membrane and wax production [95], peroxygenase 3 (*BraA03g017690.3.1C*) catalyzing hydroxylation reactions of aromatics, sulfoxidations of xenobiotics, or epoxidations of unsaturated fatty acids [96], cytochrome CYP86A4 (*BraA10g000400.3.1C*) involved in cutin biosynthesis in flower tissues [97], pectinesterases (*BraA02g008500.3.1C; BraA06g042290.3.1C*) catalyzing the hydrolysis of the methyl esters of pectin in the cell wall [98], and polygalacturonase (*BraA09g052130.3.1C*) catalyzing depolymerization of pectin in the cell wall [99]. Consequently, GB appears to coordinate a broad range of gene expression profiles in such a way as to diminish metabolic flux from primary metabolism to secondary metabolism at the young seedling stage of *B. rapa*. Down-regulation of phenylpropanoid biosynthesis and arginine/proline metabolism in GB-primed heat-stressed *B. rapa* contradicts with the previous reports that heat stress led to up-regulation of the genes associated with those metabolisms and accumulation of flavonoids in Clematis and *Capsicum annuum* plants [10]. This could be best explained by difference in growth stage at the time of heat stress because, unlike the *B. rapa* seedlings used in our study, heat stress was imposed on two-year-old Clematis and adult *Capsicum annuum* plants. Moreover, accumulation of secondary metabolites that play important roles in plant defense and storage for nutrient mobilization would be unnecessary and potentially phytotoxic if accumulated in high quantity at the young seedling stage in the absence of pathogen infection [15].

According to the KEGG enrichment results, expression of the genes for photosynthesis was not appreciably altered by GB priming, despite the well-known positive effect of GB pretreatment on photosynthesis rate [27,100,101]. This suggests that the main role of GB in increasing photosynthesis performance is to reduce excitation pressure and redox imbalance caused by photosynthetically generated electrons and sustain the integrity of photosynthetic apparatus by acting as a molecular chaperone [15]. This notion is supported by the fact that enhanced protection of photosynthesis against high temperatures in GB-accumulating transgenic tobacco plants was not due to the direct activation of PSII but to the enhancement of the activation of ribulose 1,5-bisphosphate carboxylase/oxygenase (Rubisco) by Rubisco activase to increase carbon assimilation [21,35]. In summary, GB priming coordinated adaptive cellular and metabolic responses through a complex, hierarchically organized set of regulatory networks that control the expression of antioxidant and heat shock response, as well as the pathways of MAPK signaling, phytohormone, and calcium signal transduction, and secondary metabolism.

## 4. Materials and Methods

### 4.1. Plant Materials and Treatments

The *B. rapa* variety of ‘Beijing No. 3’ was used in this study. Seeds were sown in a soil mix of peat, vermiculite, and perlite in a 3:2:1 ratio and cultivated in an incubator for 20 days under the conditions of 24 °C/22 °C (day/night), 65% relative humidity, 600 μmol·m^−2^·s^−1^ maximum light intensity of white light, and photoperiod of 16/8 h (light/dark). Uniform seedlings with three fully expended leaves were transplanted to plastic pots under the same conditions. The leaves were then sprayed daily with H_2_O as control and GB (15 mM, Beijing Solarbio Science Technology Co., Ltd., Beijing, China) for 5 days, after which all seedlings were subjected to heat stress (45 °C in 16-h light/35 °C in 8-h dark) for 5 days.

### 4.2. Measurement of RWC, Superoxide Radical and ATP Contents

Three plants per replicate were harvested and weighed immediately after removing the roots. Leaf relative water content (RWC) was determined following the method of Xu [102]. Fresh shoots were weighed quickly to obtain fresh weight (FW). Shoots were then soaked in distilled water for 4 h, and turgid weight (TW) was measured. To measure dry weight (DW), the shoots were dried at 80 °C for 24 h. RWC was calculated as follows: RWC (%) = [(FW-DW)/(TW-DW)] × 100. Superoxide radical (O_2_^·−^) was detected using the superoxide radical kit R30343 (Yuanye, Shanghai, China).

Leaf samples were used to measure ATP content. Homogenate was centrifuged for 10 min at 8000× *g* at 4 °C. Absorbance of the collected supernatant was measured with an ultraviolet spectrophotometer at 700 nm. ATP content was measured using an ATP assay kit (Solarbio, Beijing, China) and was expressed as μmol∙g^−1^ DW.

### 4.3. Total RNA Extraction and Quality Control

Total RNA was extracted from the leaves using a NEBNext UltraTM RNA Library Prep Kit for Illumina (NEB, USA) according to the manufacturer’s instructions. RNA degradation and contamination were monitored using 1% agarose gels. RNA purity was assessed using NanoDrop 2000 (Thermo Fisher Scientific, Wilmington, DE, USA). RNA integrity was evaluated using the RNA Nano 6000 Assay Kit of the Agilent Bioanalyzer 2100 system (Agilent Technologies, Santa Clara, CA, USA).

### 4.4. RNA Sequencing and RNA-Seq Data Analysis

RNA sequencing analysis was conducted by Biomarker Technologies (Beijing, China). A total amount of 1 μg RNA per sample was used as an input material for RNA sequencing. Sequencing libraries were generated using NEBNext UltraTM RNA Library Prep Kit for Illumina (NEB, USA), following the manufacturer’s recommendations, and index codes were added to attribute sequences to each sample. The adaptor sequences and low-quality sequence reads were removed from the datasets. Raw sequences were transformed into clean reads after data processing. For de novo assembly, the high-quality clean reads were mapped back to the contigs with the parameters set at a similarity of 90%. Subsequently, the contigs were assembled to construct transcripts with pair-end information and clustered to obtain unigenes. A sequence similarity search was performed against seven databases to investigate the putative functions of the unigenes based on sequence or domain alignment. All unigenes were compared with genes in the NCBI Non-redundant protein (Nr), NCBI Non-redundant nucleotide (Nt), the Protein Families Database (Pfam), Eukaryotic Orthologous Groups (KOG)/Clusters of Orthologous Groups (COG), Swiss-Prot, Kyoto Encyclopedia of Genes and Genomes (KEGG), Orthology (KO), and Gene Ontology (GO). Differential expression analysis of two conditions/groups was performed using the DESeq2. To minimize the false discovery rate of the genes detected by DESeq2, the *p* values were adjusted using the Benjamini and Hochberg’s approach for controlling the false discovery rate, and the genes with an adjusted *p*-value < 0.05 were assigned as differentially expressed genes (DEG).

### 4.5. RNA-Seq Data Validation by qRT-PCR

High quality RNA samples from the leaves of Ctrl and GB-primed seedlings were used for qRT-PCR to measure the transcriptional levels of DEG. The cDNA synthesis and qRT-PCR reactions were carried out using a HiScript^®^ Ⅲ All-in-one RT SuperMix Perfect Kit on a qTOWER3G Real-Time PCR Detection System (Analytik Jena, Jena, Germany). The house-keeping gene *BrActin* was used as a reference gene for quantitative validation of the expression data. The gene-specific primers designed for the nine selected DEGs are listed in Table S4. The reaction system was as follows: 10 μL of 2 × qPCR mix, 0.5 μL of 10 μM gene primer, 2 μL cDNA, and 7 μL of ddH_2_O. The PCR cycling conditions comprised an initial polymerase activation step of 95 °C for 30 s, followed by 40 cycles of 95 °C for 8 s and 60 °C for 6 s, and a dissociation curve analysis was carried out from 60 °C to 95 °C at the end of a PCR experiment. The 2^−ΔΔCT^ method was used to calculate the fold change of transcript level [103]. Data were analyzed from three independent sets of biological replicates with three technical replicates for each.

### 4.6. Statistical Analyses

For all treatments, five biological replications were performed. The results were expressed as mean ± standard error, and Graph Pad prism 9.0.0 software was used to draw the graphs and analyze the data. All data were subjected to analysis of variance for a factorial experiment in a completely randomized design. Statistically significant differences between means were determined at *p* < 0.05 using Tukey’s HSD (honestly significant difference) test.

## 5. Conclusions

The results from this work clearly indicated that GB priming triggered orchestrated changes in the upstream calcium sensing and hormonal signaling cascade network outputs, leading to the downstream transcriptional reprogramming of stress-related gene expression and that subsequently altered biochemical and physiological responses (ROS detoxification, HSPs/chaperon functions, osmoregulation, water and ion transport, and carbon assimilatory readjustment), which may assist plants to re-establish cellular homeostasis and acquire thermotolerance while sustaining efficient growth. Overall, GB activated ROS scavenging, heat shock response, and DNA damage repair systems, while suppressing plant immunity in the absence of biotic stress and the accumulation of secondary metabolites in juvenile *B. rapa* seedlings to increase plant fitness. This was accompanied by finely modulated co-expression of TFs associated with a complex, hierarchically organized network of calcium and hormonal signaling pathways of ABA, SA, auxin, and cytokinin in order to balance plant growth and stress responses based on the developmental and environmental cues perceived by extra- and intra-cellular receptors. GB-induced transcriptomic, proteomic, and metabolic profiles may greatly differ depending on the type of stresses as well as the stage of growth and development due to the plant’s ability to monitor and respond to the type and intensity of stresses as a controlling mechanism that integrates the influence of environmental conditions with internal developmental programs regulated by phytohormones. Therefore, more detailed investigations into the impacts of exogenous GB on *B. rapa* plants at different stages of vegetative growth are needed to gain a holistic view of flexible and dynamic processes at the molecular, physiological, and metabolic levels, thus helping to provide a comprehensive strategy for improving plant productivity through the acquired thermotolerance.

## Data Availability

The data presented in this study are available in this manuscript.

## Supplementary Materials

The following supporting information can be downloaded at: https://www.mdpi.com/article/10.3390/ijms24076429/s1.
